# SARS-CoV-2 Complicated by a Large Hemorrhagic Pericardial Effusion

**DOI:** 10.7759/cureus.22282

**Published:** 2022-02-16

**Authors:** Nardine Abdelsayed, Benjamin Mckinney, Mary Carter

**Affiliations:** 1 Department of Internal Medicine, Grand Strand Medical Center, Myrtle Beach, USA; 2 Department of Emergency Medicine, Grand Strand Medical Center, Myrtle Beach, USA

**Keywords:** covid-19, cardiac tamponade, sars-cov-2, hemorrhagic effusion, pericardial effusion

## Abstract

Severe acute respiratory syndrome coronavirus (SARS-CoV-2), primarily a respiratory virus, has also presented with cardiac complications including myocarditis, myocardial infarction, and cardiac arrhythmias. Pericardial effusions are also emerging in the literature as a sequel to this viral infection. A case of a 57-year-old Hispanic female with SARS-CoV-2 infection two months prior with worsening dyspnea on exertion who was found to have a large hemorrhagic pericardial effusion with early tamponade physiology was presented in this article. This case highlights the rare complication and the importance of bedside echocardiogram in patients with recent SARS-CoV-2 infection who present with shortness of breath and other signs of pericardial effusion.

## Introduction

Pericardial effusions can occur in the setting of several infectious and noninfectious conditions, whereas hemorrhagic effusions are usually caused by underlying malignancy, percutaneous intervention (PCI), as a complication of myocardial infarction [1], postpericardiotomy syndrome, trauma, or in the setting of mixed connective tissue disease [2]. The effusion may be transudative, exudative, or hemorrhagic depending on the etiology. In parts of the world where mycobacterium tuberculosis (TB) is prevalent, this infection also has a known association with hemorrhagic effusions. Hemorrhagic effusions are rarely associated with viral infections such as SARS-CoV-2. Our case highlights this important complication of SARS-CoV-2 and its potential morbidity and mortality.

## Case presentation

Our patient is a 57-year-old Hispanic female with a past medical history of hypertension, premature ventricular contractions, and a recent coronavirus infection two months prior who presented with two weeks of worsening shortness of breath. She had not received any of the SARS-CoV-2 vaccinations. Her shortness of breath occurred with any exertion and was associated with epigastric pressure. She also complained of mild lower extremity swelling. There was no associated dizziness, syncope, weight loss, or cough.

Her social history was notable for previously living in South Korea with her family as well as everyday vaping and alcohol use. Family history included a mother who had tuberculosis when she was a child. She did receive the Bacillus Calmette-Guérin (BCG) vaccine as a child and frequently tested positive for purified protein derivatives (PPD) but never had a personal history of active tuberculosis infection.

Vital signs on admission noted a temperature of 100.1°F, heart rate varying between 90 and 110 beats per minute (normal 60-100), blood pressure of 124/85 mmHg, and normal oxygen saturation at 98% on room air. Her physical exam was largely unremarkable except for trace bilateral lower extremity swelling.

**Figure 1:**
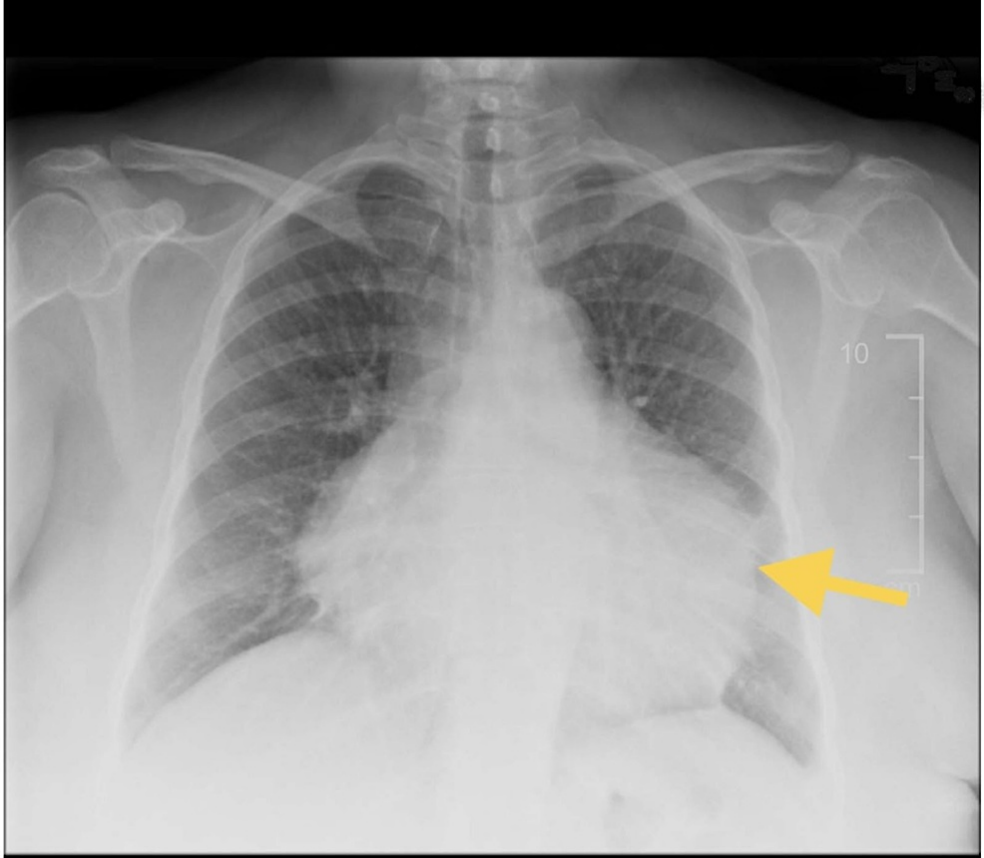
Chest x-ray anterior-posterior (AP)/posterior-anterior (PA) with the yellow arrow showing an enlarged cardiac silhouette

Laboratory findings are shown in Table 1, which are notable for normocytic anemia, thrombocytosis, and elevated liver function tests including alanine transaminase (ALT) and alkaline phosphatase (ALP). Her inflammatory markers were also elevated. Abdominal ultrasound showed hepatic steatosis. Her chest x-ray showed an enlarged cardiac silhouette (Figure 1).

**Table 1:** Abnormal laboratory findings

	Laboratory values	Reference values
Hemoglobin	10.5 gm/dl	11.6-15.4 gm/dl
Platelet count	363 k/mm^3^	156-352 k/mm^3^
Alanine aminotransferase	69 U/L	<46 U/L
Alkaline phosphatase	143 U/L	<126 U/L
Erythrocyte sedimentation rate	54 mm/hr	<20 mm/hr
C-reactive protein	4.2 mg/dL	<0.99 mg/dL

**Figure 2:**
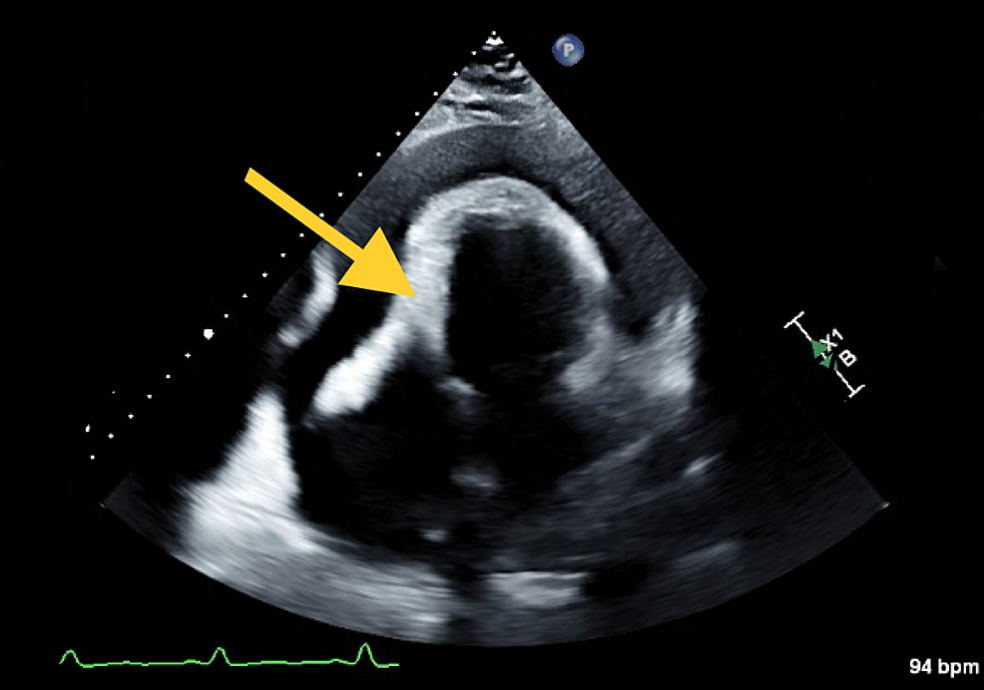
Transthoracic echocardiogram with noticeable large effusion and the yellow arrow showing the right ventricular collapse

**Figure 3:**
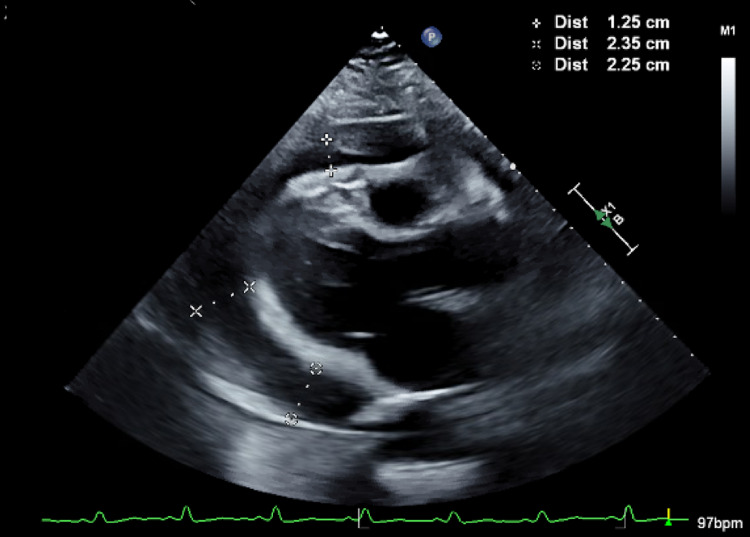
Transthoracic echocardiogram showing pericardial effusions with dimensions at the right upper corner

**Figure 4:**
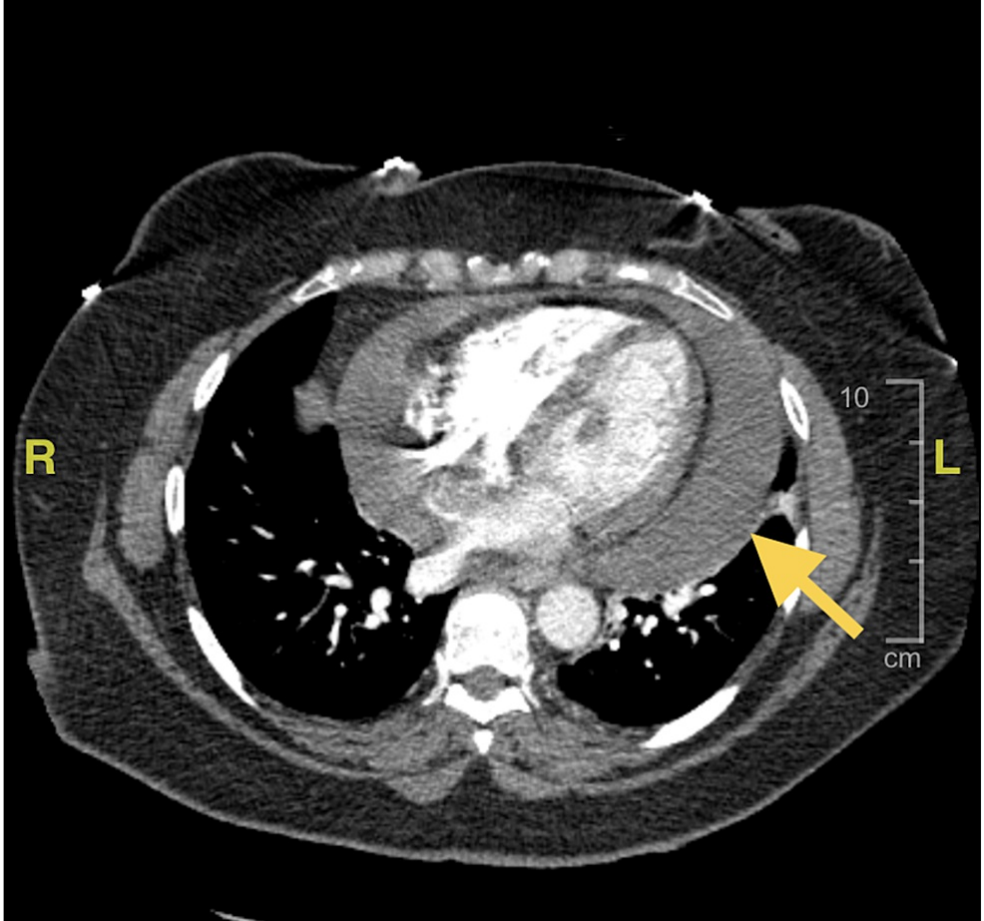
Computed tomography angiography (CTA) of the chest with the yellow arrow showing large circumferential pericardial effusion

Her electrocardiogram (EKG) noted sinus tachycardia with low voltage. Given chest x-ray and EKG findings, bedside ultrasound was performed in the emergency department, which showed a large effusion but no obvious signs of tamponade. Formal transthoracic echocardiogram showed a large pericardial effusion as well as grade 2 diastolic dysfunction and early tamponade physiology including a partially collapsed right ventricle (RV) and a mildly dilated inferior vena cava (Figures 2, 3). The effusion was later demonstrated on computed tomography angiography (CTA) chest (Figure 4).

The patient was started on intravenous normal saline at 150 cc/hr and urgently underwent pericardiocentesis with the removal of 800cc of dark serosanguinous fluid. Fluid studies showed a hemorrhagic effusion with pH 8.5, elevated white blood cell count, and an extremely elevated red blood cell count (Table 2). The fluid was predominant with polymononucleocyte (Table 3). Fluid cytology did not show any malignant cells. QuantiFERON-TB Gold test was negative, and the fluid acid-fast stain did not show any bacilli to indicate mycobacterium tuberculosis.

**Table 2:** Pericardial fluid laboratory studies

	Laboratory values	Reference values
Fluid white blood count	8 per mm^3^	0 per mm^3^
Fluid red blood cells	800,000 per mm^3^	0 per mm^3^

**Table 3:** Pericardial fluid differential

	Percent composition
Fluid polymorphonucleocytes	67%
Fluid lymphocytes	17%
Fluid monocytes	14%
Fluid eosinophils	1%

The patient tolerated the procedure well. On the next day, another formal limited transthoracic echocardiogram showed a trivial pericardial effusion, and she was discharged home on colchicine, ibuprofen, and instructions for a follow-up echocardiogram in four weeks.

## Discussion

Pericardial effusions occur in a variety of conditions. Large and hemorrhagic pericardial effusions are commonly associated with malignancy (about 26%), PCI procedures, and complications of myocardial infarctions [1]. SARS-CoV-2 has been associated with hemorrhagic pericardial effusions in recent literature [3-5]. The mechanism of the hemorrhagic effusion is hypothesized to be due to direct cardiomyocyte and pericardium invasion by binding of the angiotensin-converting enzyme-2 (ACE2) receptor that promotes inflammation, cytokine release, fibrosis, vasoconstriction, and oxidative stress in the setting of adult respiratory distress syndrome (ARDS) [6]. SARS-CoV-2 infection is initiated by viral binding to ACE2 receptors embedded in the cell membrane. Binding is performed when an S protein known as spike that is expressed in the viral coat binds to ACE2 and mediates virus uptake via a serine protease. ACE2 is found in particularly increased concentration in the lung, heart, vascular, renal, CNS, and intestinal tissues. This is hypothesized to be the reason why patients infected with SARS-CoV-2 have an increased risk of developing pericarditis, myocarditis, and/or acute coronary syndromes [7].

Pericardial fluid accumulation and subsequent cardiac tamponade may be life-threatening. Acute accumulation presents as dyspnea and chest pain and may result in cardiogenic obstructive shock. Physical exam findings include jugular venous distention, muffled heart signs, and hypotension (known as Beck’s triad). Generally, if the fluid accumulation is subacute, the symptoms are less pronounced and more insidious. Patients range from asymptomatic to gradual onset of shortness of breath, chest pain, and eventually heart failure symptoms and obstructive cardiac shock.

Electrocardiogram (EKG) can show sinus tachycardia with low voltage and possible electrical alternans, which is described as a beat-to-beat alteration in QRS complex amplitude. A chest x-ray can show an enlarged cardiac silhouette. Transthoracic echocardiogram plays an essential role in diagnosis. Tamponade presents with a collapse of the right atrium at the end-diastole and RV at the early diastole [8]. Reciprocal changes in the left and right ventricular volumes with respiration can also be seen as well as dilation of the inferior vena cava and less than 50% reduction in its diameter during inspiration.

Once the diagnosis is established, the patient who is experiencing signs of tamponade should be promptly started on intravenous fluids and definitively treated with pericardiocentesis. The procedure can be done by the emergency physician in the emergency department if immediately life-threatening or if the patient is in pulseless electrical activity (PEA) arrest. In less emergent cases, a cardiologist should be consulted to perform a pericardiocentesis under ultrasound guidance in a sterile environment. In cases where the fluid appears loculated or reaccumulates after drainage, surgical drainage can also be performed. Fluid is then analyzed with gram stain, cell count, bacterial and fungal cultures, cytology, acid-fast stain, and mycobacterial cultures to search for the inciting factor. Possible etiologies include infectious (as noted in this article), trauma, rheumatic disorders, malignancies, and amyloidosis. The underlying etiology should be treated along with drainage of the pericardial fluid. When the underlying etiology is pericarditis, non-steroidal anti-inflammatory drugs (NSAIDs) and colchicine play an important role in the treatment. Corticosteroids and anti-interleukin-1 medications (such as anakinra) can be used for refractory or recurrent cases. After monitoring for reaccumulation for 24-48 hours, patients can be discharged home with a follow-up echocardiogram in four to eight weeks.

## Conclusions

SARS-CoV-2 has presented with many cardiac complications since its emergence in 2019. A rare but important complication is hemorrhagic pericardial effusion. Early symptoms such as sinus tachycardia, chest pain, and worsening shortness of breath in patients with a remote history of SARS-CoV-2 infection should prompt a bedside echocardiogram to evaluate for possible pericardial effusion and cardiac tamponade. Prompt treatment with intravenous fluids and pericardiocentesis should then be initiated to prevent progression into cardiogenic shock.
